# A new perspective on the etiology of impacted maxillary canines: timing factors not to be ignored—a review

**DOI:** 10.1007/s44445-026-00137-z

**Published:** 2026-03-09

**Authors:** Quanquan Ren, Umi Mardhiyyah Mat Ali, Norma Ab Rahman

**Affiliations:** Orthodontic Unit, School of Dental Sciences, Universiti Sains Malaysia, Health Campus, 16150 Kubang Kerian Kelantan, Malaysia

**Keywords:** Impacted maxillary canine, Etiology, Guidance theory, Genetic theory, Timing factor, Chronological–biological mismatch

## Abstract

Impacted maxillary canine (IMC) is a common clinical problem in Dentistry and can lead to various complications. However, the etiology is complex, and no definitive conclusions have been reached to date. Traditional etiological theories cannot fully explain the mechanisms underlying IMC and often show considerable disagreement. Inspired by the clinical effectiveness of early intervention and considering the characteristics of tooth eruption and development, this study proposes a new perspective on the etiology of IMC. The concept of timing factors is proposed, and the notion of a chronological–biological mismatch is employed to explain its significance, emphasizing that it should not be overlooked. The aim is to use timing factors as a linking element to shift IMC etiological research from the traditional static, terminal-position perspective to a dynamic, process-oriented approach, and to promote the transition from conventional single-mechanism models toward a multifactorial understanding of its etiology. Clinically, early interceptive strategies, such as extraction of primary canines and rapid maxillary expansion (RME), are highly effective when applied within critical periods, highlighting the practical significance of timing factors. Recognizing developmental timing as an important etiological element provides a new perspective for studying IMC, addresses the limitations of traditional theories, and can guide precise, individualized early interventions in the future.

## Introduction

Impacted permanent teeth are a common developmental abnormality in oral clinical practice. They refer to permanent teeth that fail to erupt into the oral cavity at the appropriate time when the root of the tooth is more than 2/3 developed. Canine teeth are the most commonly impacted teeth after third molars, with impacted maxillary canines (IMC) being the most frequent type (Litsas and Acar 2011). Studies have shown that the overall prevalence of IMC is approximately 1–3% (Kalavritinos et al. 2020), varying by region and population: 0.93% in Brazil (Menezes et al. 2024), 1.10% in Sweden (Lövgren et al. 2019), 2.70% in Saudi Arabia (Alshawy 2023), and 2.96% in Egypt (Samih et al. 2021). The incidence is higher in females and often occurs unilaterally (Grisar et al. 2019; Lövgren et al. 2019; Samih et al. 2021).

If IMC is not managed and treated in a timely manner, it may lead to a series of adverse outcomes, including pain, infection, cyst formation, tooth ankylosis, and resorption of adjacent tooth roots; in severe cases, it may even result in a reduction in dental arch length (Alshehri et al. 2023). Although combined orthodontic and surgical treatment is considered an effective approach for managing IMC, delayed or improper treatment often increases the risk of complications, forcing patients to undergo more complex orthodontic-surgical interventions (Ericson and Kurol 2000). Therefore, early diagnosis and interception of potential canine impaction are regarded as the most ideal treatment strategy (Bedoya and Park 2009). Studies have shown that as patients age, both the difficulty and duration of IMC treatment significantly increase, while treatment outcomes tend to decline (Alsmnan et al. 2023). Based on this, early identification and interception of canine impaction hold important clinical significance.

Clinical evaluation combined with radiographic examination is a common approach for diagnosing IMC. Recent studies have shown that the use of cone-beam computed tomography (CBCT) can significantly improve diagnostic efficiency and accuracy (Oliveira et al. 2023). Evidence indicates that interceptive measures—such as extraction of primary teeth, rapid maxillary expansion (RME), cervical headgear, and transpalatal arch appliances—when applied early, can promote the eruption of impacted canines to varying degrees and may reduce the need for subsequent complex orthodontic and surgical treatments. Furthermore, existing evidence consistently suggests that the earlier the identification and interception of impacted canines, the more favorable the treatment outcomes, further emphasizing the critical value of early recognition and intervention in the management of IMC (Elangovan et al. 2019).

Although considerable progress has been made in the diagnosis and clinical management of IMC, its etiological mechanisms remain insufficiently elucidated, and considerable discrepancies persist among different etiological theories. This limitation, to some extent, restricts the precision and effectiveness of early prevention and interception strategies. This review aims to comprehensively summarize the main etiological theories of IMC, while introducing the time-related perspective as a novel approach to re-examine and discuss the existing evidence. By emphasizing the potential role of developmental timing in the occurrence of IMC, this review seeks to further refine the etiological framework of IMC and provide a theoretical basis for optimizing early clinical prevention and intervention strategies.

## Etiology of impacted maxillary canine

### Etiology factors of IMC

The etiology of IMC is diverse and complex and may involve a single factor or a combination of multiple factors. These factors are generally classified into systemic, local, and genetic factors. Table 1.

**Table 1:** Etiological factors of impacted maxillary canines

Etiological type	Pathogenic factors
Local factor	Local space deficiency Deciduous canine root is not absorbed Prolonged retention or premature loss of deciduous canines Ankylosis of canine Neoplasm or Cyst Excessive root curvature Lateral incisor hypoplasia Lateral incisor displacement Root interference of the first premolar Supernumerary tooth Morphological abnormalities of the canine Dental trauma/Maxillofacial trauma Apical periodontitis of deciduous teeth Premature root closure Disturbances in tooth eruption sequence Mucosal barriers- scar tissue: trauma/surgery Gingival fibromatosis/gingival hyperplasia Tooth development abnormalities Bone density differences Tooth germ rotation Long eruption path Iatrogenic factors Idiopathic factors
Systemic factors	Cleidocranial dysplasia Achondroplasia Vitamin deficiency (A/D) Amelogenesis imperfecta Osteoporosis Down syndrome Pituitary hypofunction Hypothyroidism Febrile Illness Radiation (radiation therapy)
Genetic factors	primary displacement of the tooth bud tooth germ anomaly alveolar clefts

**Related literature**

(Alqerban et al. 2016; Amuk et al. 2021; Arvind et al. 2022; Aslan and Üçüncü 2015; Becker 1995; Becker and Chaushu 2015; Bedoya and Park 2009; Bertl et al. 2013; Brin et al. 1986; Consolaro et al. 2020; Guarnieri et al. 2022; Kale et al. 2013; Mellara et al. 2014; Peck et al. 1994; Reddy et al. 2018; Rutledge and Hartsfield 2010; Sajnani and King 2012; Shapira et al. 2000; Shapira and Kuftinec 1998; Stabryla et al. 2023; Tsuji et al. 2020; Tsurumi et al. 2022).

There are many local factors involved in the etiology. It includes insufficient local eruptive space (Cacciatore et al. 2018), deciduous canine root is not absorbed, ankylosis of canine (Consolaro et al. 2020), excessive root curvature (Amuk et al. 2021), developmental anomalies of the lateral incisor (missing, microdontia, peg-shaped) and variation in root formation timing (Guarnieri et al. 2022). Root interference of the first premolar as well as supernumerary teeth (Bertl et al. 2013; Stabryla et al. 2023). Some of these factors are even thought to be related to medical interventions. For example, secondary bone grafting in patients with cleft lip and palate has been identified as a contributing factor to the occurrence of IMC (Tsurumi et al. 2022).

Numerous systemic factors may trigger IMC. It is often associated with many diseases and syndromes, including cleidocranial dysplasia (Tsuji et al. 2020), achondroplasia (Kale et al. 2013), Down syndrome (Shapira et al. 2000), and those related to endocrine issues: Pituitary hypofunction (Reddy et al. 2018), as well as some febrile diseases and radiation therapy (Mellara et al. 2014).

Purely genetic factors refer to certain special cases. For example, primary displacement of the tooth bud (Becker and Chaushu 2015), tooth germ anomalies, and alveolar clefts (Peck et al. 1994).

### Etiological theory of IMC

In early studies on the etiology of IMC, dental crowding was considered the primary cause. However, as research progressed, this view has not received consistent support. Some authors have pointed out that insufficient arch length is mainly associated with buccally impacted maxillary canines (BIMC), whereas palatally impacted maxillary canines (PIMC) are less commonly observed in crowded arches (Sajnani and King 2012). Therefore, it is generally believed that BIMC and PIMC may arise from different etiological mechanisms. The crowding or space deficiency theory is commonly used to explain BIMC. For PIMC, current literature tends to favor the guidance theory and the genetic theory as the main explanations (Bedoya and Park 2009; Counihan et al. 2013; Sajnani 2015).

The guidance theory proposes that, during normal eruption, the maxillary canine relies on the root of the adjacent lateral incisor as a “guide rail” for both spatial positioning and eruption direction. When the lateral incisor is absent or presents with developmental anomalies such as agenesis, microdontia, a conical shape, or abnormal root formation, this guiding mechanism may be disrupted, resulting in deviation of the canine eruption path and an increased risk of palatal displacement or impaction (Becker and Chaushu 2015; Becker et al. 1981). This theory is supported by epidemiological observations showing a higher prevalence of anomalous maxillary lateral incisors in patients with palatally displaced or impacted canines (Kim et al. 2017; Oliveira et al. 2023; Yan et al. 2013).

However, in contrast to the guidance theory, which focuses on local mechanical influences, the genetic theory proposes that IMC, particularly palatal impaction, is not primarily caused by local mechanical factors but rather represents a manifestation of abnormal genetic regulation during tooth development. Within this theoretical framework, palatally displaced canines are regarded as a hereditary dental developmental anomaly and frequently associated with other developmental abnormalities, such as tooth agenesis, dental malformations, or ectopic eruption (Devi and Padmanabhan 2019; Lupinetti et al. 2022; Peck et al. 1994). Evidence supporting the genetic theory is mainly derived from observations of familial aggregation, its association with other genetically influenced dental anomalies, and sex-related differences in prevalence (Chung et al. 2011; Peck et al. 1994).

## Considerations on the etiological theories of IMC

### Controversies and limitations of traditional theories

The guidance theory and the genetic theory constitute the two main, and to some extent competing, frameworks for explaining the etiology of IMC. As a result, they are often discussed separately, yet each has its own notable limitations. Although Becker has conducted extensive research to promote and elaborate on the guidance theory and provided substantial supporting literature, the theory still cannot fully account for certain clinical observations. For example, some studies have reported cases in which a maxillary canine erupts normally adjacent to a malformed lateral incisor on one side, while impaction occurs adjacent to a normal lateral incisor on the contralateral side (Sajnani and King 2014). Additionally, in cases with missing lateral incisors, some maxillary canines become impacted, whereas others erupt normally (Laganà et al. 2018). In light of these phenomena that the guidance theory cannot explain, many scholars argue that the genetic theory may better account for the true etiology of IMC.

Although the genetic theory proposes that IMC represents a dental anomaly with a hereditary component, supported by familial aggregation and its association with other dental anomalies, this perspective continues to exert significant influence in contemporary discussions of etiology. However, as Becker noted, genetic factors would typically be expected to exhibit pronounced bilaterality and symmetry. If IMC were entirely genetically determined, how could we explain the higher prevalence of unilateral cases? Moreover, in cases with a missing or malformed lateral incisor, why do some maxillary canines become impacted while others erupt normally? These phenomena appear inconsistent with typical genetic patterns (Becker and Chaushu 2015). Moreover, a study of monozygotic and dizygotic twins found very low concordance of canine impaction (Camilleri et al. 2008). Therefore, genetic susceptibility alone cannot account for the key clinical features of IMC, such as unilateral presentation, variation in eruption timing, and differential responses to interceptive treatment. Even among individuals with similar genetic backgrounds, eruption outcomes of the canines may vary considerably.

### Contributions and gaps of the sequential theory

These classical theories have generated considerable academic debate and divergent viewpoints when discussed in isolation. Based on the patterns of tooth eruption, some scholars proposed the sequential theory, regarding the genetic theory as the primary early determinant of IMC, while the guidance theory serves as a local auxiliary factor that establishes the direction of impaction at later stages (Sajnani and King 2012). Although they recognized the limitations of the traditional theories and attempted to integrate both to explain the etiology of IMC, this approach did not resolve the issues inherent in the traditional frameworks and still overlooked the important element, the timing factor that links the two theories.

## Etiology of IMC from a new perspective

### Critique and inspiration from a new perspective

From a clinical perspective, the essence of the crowding theory is that the adjacent teeth or the surrounding alveolar bone fail to provide sufficient eruption space for the canine. In theory, as long as adequate space is provided, the canine should be able to erupt normally. However, in practice, this is not the case. Karashima et al. reported that ages 7–8 represent the most effective period for early intervention of IMC using rapid maxillary expansion (RME), and their data demonstrated that the effectiveness of RME in addressing BIMC decreases with increasing age (Harada-Karashima et al. 2021). In addition, the extraction of primary canines is also considered an effective method to provide eruption space. However, Cobourne et al. clearly indicated that ages 10–11 are the optimal time for interceptive extraction of primary canines to manage IMC, and the success rate of such interventions decreases significantly after age 14 (Cobourne et al. 2025). These findings suggest that the mere availability of space does not determine the ultimate eruption outcome of the canine; rather, the clinical significance of space appears to depend on the developmental timing at which it is obtained.

The traditional guidance theory regards the lateral incisor as an “eruption beacon,” and studies by Becker et al. indicated that, although some lateral incisors are small, they can still provide important guidance for canine eruption (Becker et al. 1981). This essentially assumes that the mere presence of the lateral incisor automatically confers guidance function. However, Ristaniemi et al. (2022), based on a large-sample retrospective study, found that incomplete root development of the lateral incisor not only failed to guide the canine but was also significantly positively associated with the risk of subsequent treatment (Ristaniemi et al. 2022). The guiding role of the lateral incisor appears to be time-sensitive, depending on whether root development coincides with the critical stages of canine eruption and migration. This suggests that the “guidance” function of the lateral incisor is not static, but highly dependent on the timing of root development and the dynamics of spatial occupancy. Ignoring developmental timing, the guidance theory confuses anatomical presence with functional capability, committing a structure–function error.

Although Mercuri et al. found that PIMC is significantly associated with peg-shaped lateral incisors, underdeveloped lateral incisors, and inverted teeth, with several-fold increased risk, this result was previously considered to support a genetic determination of IMC (Mercuri et al. 2013). However, Camilleri et al. (2008), in a twin study, reported cases of unilateral impaction in monozygotic twins, indicating phenotypic discordance, which further supports the role of non-genetic factors in phenotypic expression (Camilleri et al. 2008). Moreover, a 2003 study on preventive treatment in monozygotic twins found that after extraction of both primary canines, one canine in each patient erupted spontaneously, but on opposite sides (Leonardi et al. 2003). These observations suggest that genetic factors may confer susceptibility rather than determine the outcome, and that phenotypic expression is modulated by the interaction of developmental timing and environmental factors during growth.

### The non-negligible timing factor

**Fig. 1:**
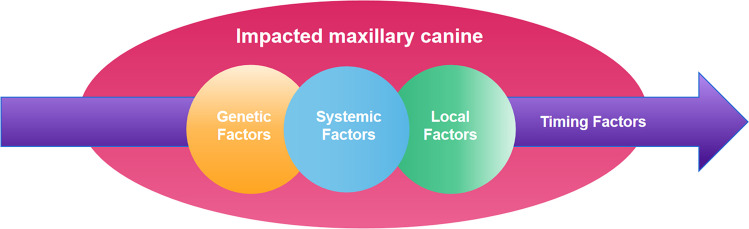
IMC etiological model incorporating timing factors

The essence of IMC lies in abnormal tooth development and eruption. This suggests that it is not merely a positional anomaly, but a disturbance in a dynamic developmental process. Genetic theory, guidance theory, and local factors should not be regarded as competing or mutually exclusive explanations for IMC. Rather, their etiological relevance is contingent upon the developmental timing at which they operate. Within specific developmental windows, these mechanisms may meaningfully influence the eruption path of the maxillary canine; outside such windows, their explanatory and corrective power becomes markedly limited. Once a critical window is missed, identical factors may no longer be sufficient to induce displacement or restore normal eruption. This perspective suggests that attempts to universally explain all clinical presentations of IMC through a single etiological theory risk overlooking a fundamental dimension of the condition—the indispensable role of developmental timing Fig. 1.

### Rationale for considering the timing factor

The theoretical basis for the timing factor is derived from the biological characteristics of the maxillary canine, the concept of timing displacement, and supporting evidence from the literature.

The maxillary permanent canine must move from the orbital floor to the occlusal plane by approximately 22 mm, representing the longest eruption path in the permanent dentition (Sajnani 2015). This extended path makes the eruption process highly dependent on timing. At age 5, the cusp tip of the canine is 21–22 mm from the occlusal plane; over the subsequent 7 years, the normal eruption group descended approximately 18 mm, whereas the impacted group descended only 3.3 mm (Sajnani 2015; Sajnani and King 2012), indicating that eruption speed is closely related to timing factors. By age 7½–8, two-thirds to three-quarters of the lateral incisor root development is complete, forming a “guiding plate threshold”; if root length is insufficient, the canine loses a stable path for descent (Becker and Chaushu 2015), demonstrating that the eruption trajectory depends on specific developmental timing. By ages 9–10, the canine completes self-correction along the distal surface of the lateral incisor root and stabilizes its direction, marking the end of the guidance phase (Becker and Chaushu 2015; Sajnani 2015). In summary, the canine’s extended eruption path and the precise alignment with the two critical timing windows; vertical movement at age 5 and guidance plate readiness at 7½–8 years highlight the central role of timing in normal eruption and emphasize the physiological significance of developmental timing.

Becker (2015) implicitly highlighted the concept of “timing discrepancy,” noting that “the timing discrepancy that is seen in the normal development of anomalous adjacent teeth has been shown to be implicitly linked with canine impaction.” His observation suggests that the existence of timing factors (developmental timing) is real and not merely hypothetical (Becker and Chaushu 2015).

The theoretical basis for the importance of timing factors stems from two lines of evidence. First, twin studies have shown that while ectopic eruption of maxillary canines is under single-gene dominant control, phenotypic expression may still vary due to external factors even among individuals with identical genetic backgrounds (Camilleri et al. 2008). This finding explains why IMC has a genetic component, yet early interceptive treatment can still be effective. Second, the “sequential hypothesis” proposed by Sajnani and King (2012) suggests that canine impaction results from the combined effect of genetic potential and the guiding influence of adjacent teeth, such as lateral incisors, providing a new perspective for understanding the mechanism of impaction (Sajnani and King 2012). These findings provide a theoretical basis for the significance of timing factors, supporting the notion that developmental timing plays a critical role in IMC and is an essential factor for understanding its pathogenesis.

From a clinical perspective, early intervention for IMC has been shown to be effective, with some regions implementing early interceptive measures achieving a notable reduction in prevalence (Lövgren et al. 2019). This indicates that genetically influenced IMC is not entirely predetermined by heredity. Furthermore, the literature highlights that interceptive strategies such as rapid maxillary extension (RME) and extraction of primary canines exhibit clear time-dependent effects. These observations suggest that both the development and correction of IMC occur within specific timing windows. From a clinical standpoint, this provides a rationale for considering the timing factor as a critical element that should not be overlooked (Benson et al. 2021; Ceraulo et al. 2025; Cobourne et al. 2025).

**Fig. 2:**
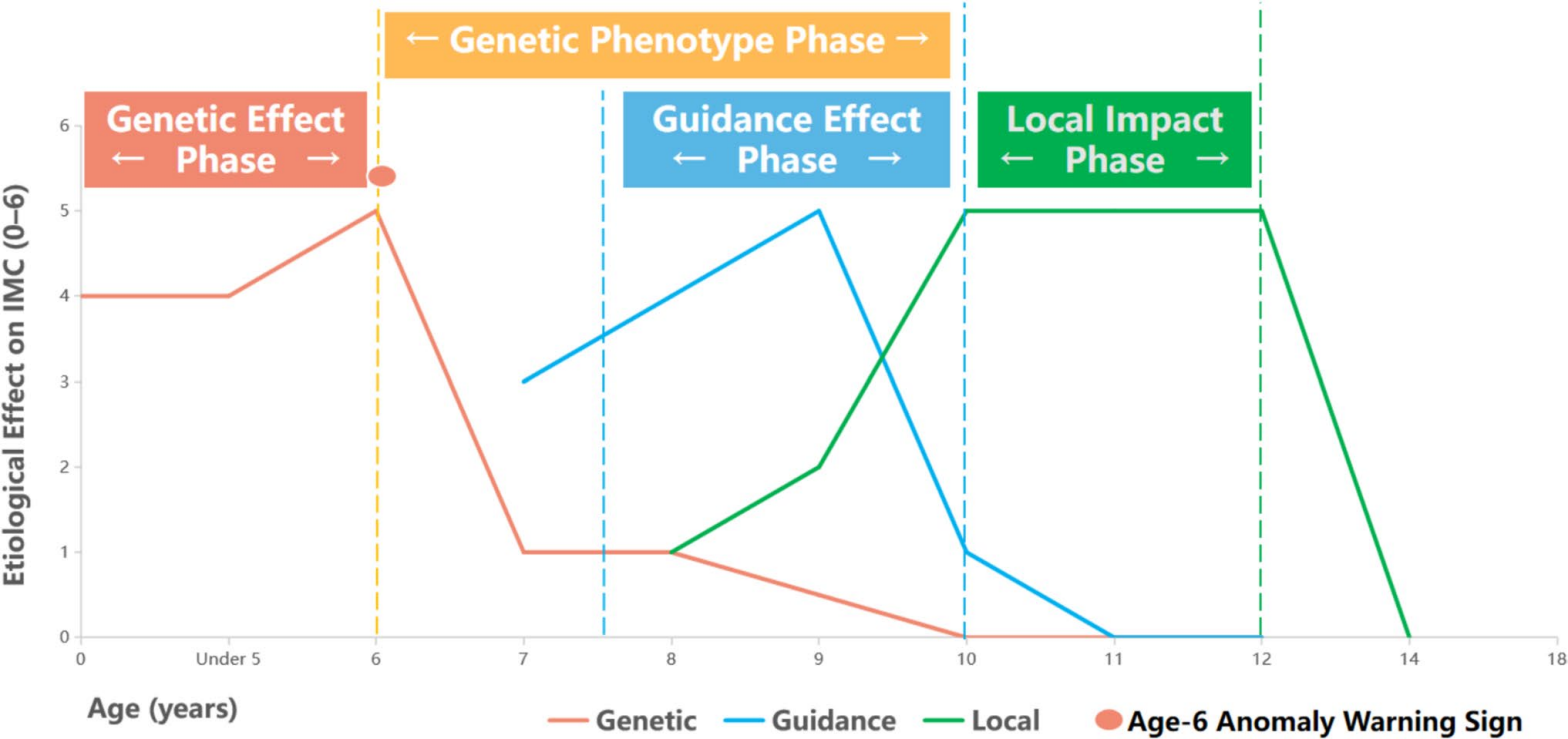
Characteristics of traditional IMC etiological theories under timing factors

Based on the aforementioned multifaceted information and evidence, it is clear that timing factors objectively exist and play a crucial linking role. Their presence allows for a better understanding of the characteristics of traditional theories, effectively transforming what would otherwise be a static debate over outcomes into a dynamic process analysis. The schematic diagram categorizes the contribution to MIC occurrence into levels 0–6, where 0 indicates no effect, 1 a very mild effect (clinically negligible), 2 a mild effect, 3 a moderate effect, 4 a strong effect, 5 a very strong effect, and 6 represents extreme cases (primary impaction). Moreover, a marker at age 6 is set as a genetic warning point — indicate abnormalities in the lateral incisors at this age strongly indicate a genetic predisposition to IMC. Along the temporal dimension, the effects of the three classical theories are clearly illustrated, and their characteristics are annotated with timing windows according to clinical features Fig. 2; sources in Table 2.

**Table 2:** IMC three classical theories: clinical timing and efficacy analysis

Force Type	Onset/Start (Age)	Peak/Critical Interceptive Window	End/Diminished Effect (Age)
Genetic Factors	**Embryonic Stage** (tooth germ patterning)	**6—9 Years** (6-yr warning sign; Optimal Period for RME)	**Post-10 Years** (Genetic phenotype established; spontaneous correction unlikely)
Guidance Effect	**7.5—8 Years** (The lateral incisor roots begin to serve a guiding role)	**9—10 Years** (Critical period for clinical assessment; radiographic evaluation optional)	** ~ 11 Years** (Canine crown passes mid-lateral incisor; physical guidance stage completed)
Local Interference	** ~ 8 Years** (Onset of hard tissue obstructions: supernumeraries or odontomas)	**10—13 Years** (Optimal period for primary canine extraction; peak interference from inflammation)	**Post-14 Years** (Canine apex closes; biological eruption force is lost; becomes permanent impaction)

**Related literature**

(Becker and Chaushu 2015; Bedoya and Park 2009; Benson et al. 2021; Ceraulo et al. 2025; Cobourne et al. 2025; Counihan et al. 2013; Elangovan et al. 2019; Harada-Karashima et al. 2021; Ristaniemi et al. 2022; Sajnani 2015; Sajnani and King 2012).

## The significance of the timing factor

In this context, the timing factor does not refer to “faster or slower development” or simply to a “longer or shorter time,” but rather to a chronological–biological mismatch. In other words, the maxillary canine follows a relatively stable developmental rhythm, whereas the developmental timing of adjacent teeth particularly the lateral incisors is highly variable. These processes must be coordinated and synchronized within a critical developmental window. Once this window is missed, even the presence of anatomical structures capable of guiding canine eruption loses its guiding function. This concept provides a compelling explanation for long-standing challenges in traditional theories, including why IMC can occur adjacent to otherwise normally developing canines and why unilateral IMC is more prevalent. It also opens new avenues for etiological research into IMC, fills an important conceptual gap, and broadens current thinking.

Moreover, the concept of chronological–biological mismatch helps address a long-standing clinical puzzle: why early interceptive extraction of primary canines for IMC sometimes yields favorable outcomes and sometimes does not. As research in this area advances, this concept may guide more precise interceptive strategies in clinical practice, thereby contributing to the effective prevention of IMC. This emphasizes the clinical relevance of timing factors in the prevention and management of IMC.

After establishing an IMC etiology model that includes the 'timing factor', to facilitate further understanding of the formation process of IMC under the 'timing factor', this review integrates the eruptive and developmental timing characteristics of the canines with the clinical characteristics of effective interception of IMC. Based on a comprehensive literature analysis, a table was created to provide a quick understanding of the IMC etiology model under the 'timing factor' and to analyze the clinical timing and efficacy under the classic IMC theories, which can guide early clinical intervention. This is intended to assist in the implementation of early clinical intervention for IMC (see Table 2 for details).

## Limitations and future directions

The timing factor proposed in this study remains at a hypothetical stage. Its theoretical basis arises from existing controversies and gaps in current etiological research, as well as relevant clinical observations from studies on the prevention and management of IMC, and is further supported by the biological characteristics of canine growth and development. Most available longitudinal studies have focused on short-term clinical interventions (such as deciduous canine extraction or maxillary expansion) and lack continuous follow-up across the entire growth period. This limitation hampers the establishment of clearly defined timing markers and critical threshold indicators across different developmental stages of the maxillary canine. Future research should therefore conduct long-term, continuous longitudinal follow-up in patients with familial IMC as well as in normal controls, to elucidate the specific roles of timing factors at different developmental stages and their interactions with other relevant variables, thereby providing a stronger foundation for the development of more precise and effective predictive and preventive models for IMC.

## Conclusion

This review provides a comprehensive overview and analysis of the traditional theories on the etiology of IMC, revealing that no single theory can fully explain its mechanism of occurrence. By re-examining these traditional theories from the new perspective of timing factors, the concept of chronological–biological mismatch is proposed as an explanation for the limitations of conventional etiological models. The review emphasizes that the timing factor should not be overlooked in favor of pursuing a single, independent cause to fully account for IMC. IMC represents a developmental and eruptive anomaly of the maxillary canine influenced by multiple factors, with timing serving as the key linking element. This characteristic not only makes early clinical intervention feasible but also, with further study, has the potential to provide guidance and a foundation for personalized, precise interceptive strategies in clinical practice.

## Data Availability

The data supporting the findings of this review are available in publicly accessible repositories, as cited in the references section of this article.
